# Social Determinants of Health Predict Sleep–Wake Disturbances Among Patients Living With Primary Brain Tumors: A Cross‐Sectional Analysis

**DOI:** 10.1002/cam4.70693

**Published:** 2025-02-15

**Authors:** Michelle L. Wright, Hope Miller, Elizabeth Vera, Alvina A. Acquaye‐Mallory, Brayden Chavis, Anna Choi, Ewa Grajkowska, Tricia Kunst, Morgan Johnson, Zuena Karim, Bennett McIver, Madhura Managoli, Jennifer Reyes, Terri S. Armstrong, Amanda L. King

**Affiliations:** ^1^ Neuro‐Oncology Branch National Cancer Institute, National Institutes of Health Bethesda USA; ^2^ Office of Patient‐Centered Outcomes Research National Cancer Institute, National Institutes of Health Bethesda USA

**Keywords:** brain tumors, financial toxicity, sleep–wake disturbances, social determinants

## Abstract

**Introduction:**

Sleep disturbance (SD) and sleep‐related impairment (SRI) significantly impact the lives of primary brain tumor (PBT) patients. We aimed to describe the prevalence of SD and SRI in this population, determine the reliability of the PROMIS‐SD and PROMIS‐SRI instruments, and identify predictive factors to support the development of targeted interventions for at‐risk individuals.

**Methods:**

This cross‐sectional study evaluated SD and SRI in PBT patients enrolled in a Natural History Study who completed 1‐year follow‐up questionnaires (*N* = 229). Demographic, clinical, and socioeconomic data were analyzed to identify factors associated with SD and SRI. Descriptive statistics were used to report the prevalence of sleep problems, and linear regression analysis was conducted to identify predictive factors. The reliability of sleep‐related instruments was calculated using Cronbach's alpha.

**Results:**

Fifteen percent of PBT participants reported clinically significant SD and 20% reported clinically significant SRI, which were associated with financial toxicity (*p* < 0.001), being unemployed (*p ≤* 0.02), and taking psychotropic medication (*p ≤ 0.*002). Good internal consistency was demonstrated by the SD (0.923) and SRI (0.925) questionnaires in this population.

**Conclusions:**

In this study, social factors such as financial toxicity and employment status were associated with SD and SRI. Psychotropic medications also impacted SD and SRI in PTB survivors, but less so than financial toxicity. Social factors and other medications may impact sleep more strongly in PBT survivors than their previous treatment courses.

**Implications for Cancer Survivors:**

SD and SRI can be impacted by multiple factors, including those not related to PBT treatment, which should be considered by their providers.

## Graphical abstract

Sleep–wake disturbances are common among primary brain tumor patients. Social factors like financial toxicity and other medications, including psychotropic medications, may impact sleep more strongly when patients are more distal from diagnosis than their previous treatment course.

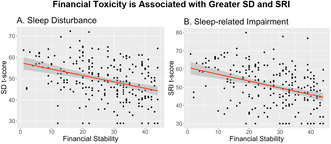

## 1 Introduction

Patients with primary brain tumors (PBTs) often have a poor prognosis and complex illness trajectory that is characterized by a high symptom burden from the time of diagnosis throughout their illness trajectory [1, 2, 3]. Some of the most reported severe symptoms PBT patients experience are sleep–wake disturbances, which can be defined as perceived or actual alterations in a person's sleep that result in impaired daytime functioning [2, 4]. These disturbances can occur during two distinct periods, including the sleep period (sleep–wake) and the wake period (activity–rest) and can result in patients reporting difficulty falling or staying asleep, waking too early or feeling unrefreshed in the morning, and having daytime sleepiness that disrupts their ability to function [5]. While sleep–wake disturbances are common and occur in approximately 10%–20% of the general population [2], they are far more common among cancer patients, with the prevalence ranging from 25% to 80%, depending on the population and timepoint of measurement [6, 7, 8]. The prevalence of sleep–wake disturbances within the PBT population ranges from 17% to 65% [1, 9], with past work demonstrating that this symptom occurs across the disease continuum from the time of diagnosis, throughout treatment, and for some individuals, years afterwards [3]. Adverse health effects related to poor sleep quality are well‐documented for cancer patients [10], including poor cognitive function, increased fatigue, pain, and irritability, mood disorders, obesity, diabetes, and poor cardiovascular health [11, 12, 13, 14], which highlights the need for the development of efficacious interventions.

There are several potential contributors to the development of sleep–wake disturbances in PBT patients. There can be direct tumor or treatment‐related effects on sleep‐sensitive areas of the brain, including the suprachiasmatic nucleus within the hypothalamus and the frontal lobe, which, when damaged, can contribute to sleep problems [15, 16]. Cranial radiation is a well‐known risk factor for the development of sleep–wake disturbances in PBT patients [2]. For example, in malignant glioma patients, a somnolence syndrome characterized by fatigue, excessive drowsiness, and concentration problems was identified during and immediately following radiation treatment [2, 17]. Additionally, the use of concomitant medications to manage disease‐ and treatment‐related symptoms, including anticonvulsants or corticosteroids, has been shown to significantly impact sleep quality, mood, and daytime hypersomnolence [18, 19]. There are also individual factors that can predispose PBT patients to sleep–wake disturbances, including pre‐existing sleep disorders [19], sleep chronotype [20], genetic polymorphisms associated with circadian regulation [21], presence of comorbid conditions [2, 22], co‐occurring mood disorders [14, 22], and poor sleep hygiene behaviors [3].

A growing area of research focuses on the impact that upstream social and environmental factors can have on sleep quality for the general population, including socioeconomic position (SEP), work and occupation, and neighborhood quality [23]. Studies examining the association between sleep quality and SEP have widely reported that individuals of lower SEP are more likely to report higher levels of sleep–wake disturbances, compared to those with more affluence [14, 24, 25, 26, 27]. Job loss and unemployment, which can significantly impact SEP, have also been shown to affect sleep quality. In a large European cross‐sectional study of over 24,000 adult workers, employment insecurity increased the odds of reporting insomnia or general sleep difficulties by 47% [28]. Similarly, a study conducted during the first year of the COVID‐19 pandemic showed a significant relationship between reported financial hardships and moderate–severe sleep disturbances, with the strongest associations reported for Black/African American individuals [29]. Neighborhood factors, such as excessive environmental noise and light levels at night, high crime, and low social cohesiveness, have also been implicated in sleep disturbances for adults and children in the United States [23, 30, 31]. While there are no known studies examining the impact of social/environmental factors on sleep quality in PBT patients, a small cross‐sectional analysis of mixed solid tumor patients showed that approximately 18% of survivors experienced high levels of financial toxicity, which was significantly associated with decreased health‐related quality of life and higher severity of affective symptoms [32].

Despite all that is known about the prevalence and contributing factors for sleep–wake disturbances in PBT patients, identifying at‐risk patients prospectively remains challenging, and there is a need to better characterize the types of sleep problems that exist within this population. The primary aim of this study was to describe the prevalence of sleep–wake disturbances in PBT patients, including sleep disturbance (SD) and sleep‐related impairment (SRI), while also establishing the reliability of sleep‐related patient‐reported outcome (PRO) instruments in this population. A secondary aim was to identify important predictors for the occurrence of SD and SRI for PBT patients, including demographic, clinical, and socioeconomic factors, so that points of intervention may be identified and targeted to improve clinical outcomes.

## 2 Methods

For this cross‐sectional study, participants were identified from the Neuro‐Oncology Branch Natural History Study (NOB‐NHS; NCT #: NCT02851706; PI: Terri S. Armstrong), which is an Institutional Review Board approved, longitudinal, observational trial that follows individuals with primary brain and spine tumors over the course of their disease. Participants must be 18 years or older to enroll in the NOB‐NHS and are eligible at any time during their disease trajectory. After obtaining written informed consent, the NOB‐NHS staff collect PROs and clinical data. Data collection began in September 2016 and is ongoing, with over 1200 patients enrolled to date. Sleep‐related PROs questionnaires were added to the NOB‐NHS trial in the summer of 2023. All active NOB‐NIH participants are required to remotely complete an annual assessment sent via email link by the NOB‐NIH staff, and responses are automatically recorded into a secure database. Only participants with PBTs that completed their 1‐year follow‐up assessments, including sleep‐related PROs, who had a confirmed tissue diagnosis were included in this analysis (*N* = 229).

### 2.1 Sociodemographic and Clinical Information

Key patient‐reported demographic and socioeconomic data, along with clinician‐reported clinical data, that were collected for the NOB‐NHS were used in this analysis. These data included age, sex, race, ethnicity, employment status, education level, income, months from diagnosis, tumor type, tumor grade, tumor location (number of sites), tumor recurrence, active treatment status, number of previous tumor treatments, number of tumor progressions, medications, and treatment history. Karnofsky Performance Score (KPS) was used to assess functional status, and age‐adjusted Charlson Comorbidity Index (CCI) was used to assess the impact of comorbid conditions on health status.

### 2.2 Patient‐Reported Outcomes

#### 2.2.1 Sleep–Wake Disturbances

To assess sleep–wake disturbances, participants completed the PROMIS‐Sleep Disturbance (SD) 8b and PROMIS‐Sleep‐Related Impairment (SRI) 8a instruments. The 8‐item SD questionnaire measures sleep restoration, perceived quality and depth of sleep, as well as perceived difficulties falling or staying asleep [33]. The 8‐item SRI measures levels of alertness, sleepiness, and tiredness during waking hours, and perceived functional impairments associated with sleep issues [33]. Using these two measures allows us to assess how our participants perceive their sleep quality through the night (SD), as well as how they perceive their sleep quality impacting their functioning during the daytime (SRI). SD and SRI raw scores are summed and converted to *T*‐scores to interpret and analyze PROMIS SD and SRI measures. *T*‐scores are rescaled raw values that allow for comparison to a general population mean of 50 and SD of 10. Participants with higher *T*‐scores have more severe SD and SRI. For example, a score of 60 on either measure would indicate the participant is 1 standard deviation above the population mean. *T*‐scores of 60 or greater were identified as individuals with clinically significant SD and SRI [33].

#### 2.2.2 Financial Toxicity

Financial toxicity among participants was measured using the Comprehensive Score for Financial Toxicity (FACIT‐COST) measure, developed among cancer patients in the United States [34]. This instrument was created to capture the amount of financial distress experienced by patients with cancer. It contains 11 items assessing financial concerns over the past 7 days with severity ranging from 0 (“none at all”) to 4 (“very much”). The score can range from 0 to 44, with lower scores indicating more financial toxicity.

### 2.3 Statistical Analysis

Descriptive statistics were used to report participant demographics, clinical and socioeconomic characteristics, and questionnaire scores. Normality was assessed, and parametric tests were used. Cronbach's alpha was calculated to assess the reliability of the sleep measures (SD and SRI). To identify demographic, clinical, and socioeconomic characteristics associated with SD and SRI, univariate regression models were completed and variables with *p*‐values < 0.05 were considered significant. Categorical variables were dummy coded for pairwise comparisons. Statistical analyses were conducted with IBM SPSS Version 25.0.41 [35].

## 3 Results

### 3.1 Sample Characteristics

Demographic and clinical characteristics for the sample are outlined in Table 1. In summary, our sample was primarily middle‐aged [median 47 years, range (20–79)], non‐Hispanic (90%), and White (80%), with slightly more male participants (57%). A majority of participants had obtained a bachelor's degree or higher (76%), were employed or retired (79%), had some form of health insurance (98%), lived with another person (83%), and were married (66%). The median age at the time of brain tumor diagnosis was 39 years old (range 5–76) with a median time from diagnosis of 65.5 months (range 0–444). Glioblastoma, astrocytoma, and oligodendroglioma were the most common tumors in our patients, with 62% being high‐grade tumors (WHO grade 3 or 4). Although most patients (83%) were not currently receiving treatment at follow‐up, the majority previously had surgery (95%), radiation (78%), or other tumor treatments (66%), with 61% having prior recurrence and 11% having radiographic progression. Over half of our participants were currently taking anticonvulsive medications (56%), with fewer participants taking psychotropic medications (27%) or corticosteroids (10%).

**TABLE 1:** Participant characteristics (*N* = 229).

Characteristic	*N*	%
Race		
Asian	11	5
Black or African American	18	8
White	184	80
Other^a^	11	5
Rather not say	5	2
Ethnicity		
Not Hispanic or Latino	206	90
Hispanic or Latino	18	8
Prefer not to answer	5	2
Sex at birth		
Female	98	43
Male	131	57
Marital Status		
Married	152	66
Unmarried	77	34
Employment status		
Employed	148	65
Retired	31	14
Unemployed	50	22
Education level		
Some college or less	55	24
Bachelor's degree	76	33
Advanced degree	98	43
Household income		
Less than $49,000	39	17
$50,000–$149,999	80	35
More than $150,000	74	33
Prefer not to answer	39	16
Health insurance		
Private	159	69
Government	66	29
None/Other	4	2
Live alone		
No	191	83
Yes	38	17
Current diagnosis		
Astrocytoma	64	28
Ependymoma	13	6
Glioblastoma	38	17
Meningioma	12	5
Oligodendroglioma	53	23
Other	49	21
Tumor grade level		
Low grade	80	35
High grade	142	62
None assigned	7	3
Active treatment		
No	189	83
Yes	40	18
Surgery treatments		
1	135	56
2 or more	94	39
Radiation treatments		
None	54	23
1	158	66
2 or more	28	12
Other treatments (non‐surgical or non‐radiation)		
None	81	34
1	113	47
2 or more	46	19
Radiographic progression		
No	204	89
Yes	25	11
Prior recurrence		
No	150	63
Yes	90	38
Number of recurrences		
None	150	63
1	40	17
2 or more	50	21
Original surgery extent		
Biopsy	24	11
Subtotal	84	37
Gross total	96	42
Resection NOS	25	11
Number of tumor sites		
Single	192	84
Multiple	37	16
Location		
Supratentorial	200	87
Infratentorial	26	11
Both	3	1
KPS		
60	7	3
70	8	4
80	23	10
90	100	44
100	77	34
Not assessed	14	6
CCI age adjusted		
0	100	44
1	46	20
2	20	9
3	9	4
4	4	2
5	1	< 1
Missing	49	21
Anticonvulsant use		
No	94	41
Yes	127	56
Not assessed	8	4
Corticosteroid use		
No	199	87
Yes	22	10
Not assessed	8	4
Psychotropic use		
No	160	70
Yes	61	27
Not assessed	8	4

^a^ Race Other: Asian American, Brazilian, Hispanic, Indian, Indo‐Iranian, Korean/American, Latin, Middle Eastern, Puerto Rican, Slavic/Hispanic/Scandinavian, White/Pacific Islander.

### 3.2 Main Findings

Clinically significant SD and SRI were reported by 15% and 20% of our participants, respectively, with 8% endorsing both SD and SRI (Table 2). Cronbach's alpha scores indicate good reliability for assessing SD (0.923) and SRI (0.925) among our PBT participants.

**TABLE 2:** Summary of sleep measures (*N* = 229).

	Mean, SD	Median, Range
PROMIS SD *T*‐Score	49.3, 9.4	49.0, 28.9–72.3
PROMIS SRI *T*‐score MEQ score	50.8, 10.7 57.9, 9.6	51.0, 30.0–79.9 59, 24–78
	*n*	%
Clinically significant SD	34	15
Clinically significant SRI	46	20
Clinically significant SD and SRI	20	8

**FIGURE 1:**
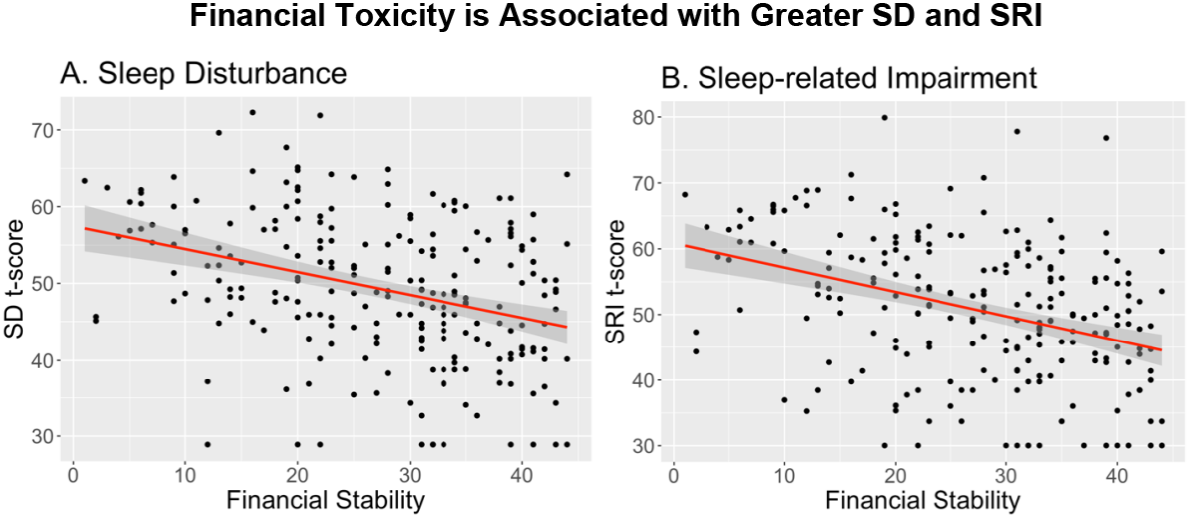
Impact of financial toxicity on SD and SRI. Zero on the scale of financial toxicity indicated the highest level of toxicity, and the greater the number, the less toxicity an individual experiences. Here, as the amount of toxicity decreases, the level of SD and SRI also decreases.

Univariate analysis revealed that financial well‐being (*β* = −0.30, 95% CI [−0.41, −0.19]), as measured by the FACIT‐COST, and not having an assigned tumor grade (*β* = −7.90, 95% CI [−15.3, −0.66]), were both associated with lower SD scores. Being unemployed (*β* = 3.95, 95% CI [0.96, 6.94]), and taking psychotropic medications (*β* = 5.16, 95% CI [2.47, 7.85]) were associated with more SD. The financial well‐being, or toxicity, experienced by participants had the largest effect on SD scores (*R* ^2^ = 0.12), followed by the use of psychotropic medications (*R* ^2^ = 0.06), employment status (*R* ^2^ = 0.03), and no assigned tumor grade (*R* ^2^ = 0.02). Participants with higher financial well‐being also reported less SRI (*β* = −0.37, 95% CI [−0.49, −0.25]). Among our participants, being unemployed (*β* = 4.07, 95% CI [0.66,7.49]) and taking psychotropic medications (*β* = 4.67, 95% CI [1.58,7.75]) was also associated with more SRI. The effect of financial well‐being (*R* ^2^ = 0.14), psychotropic medications (*R* ^2^ = 0.04), and employment (*R* ^2^ = 0.03) on SRI was similar in magnitude to those observed with SD. Univariate results for all variables assessed are reported in Table 3. When we visualize results that had the greatest effect, financial toxicity had a significant negative impact on both SRI and SD of similar magnitude (Figure 1).

**TABLE 3:** Potential predictors of SD and SRI.

Variable	SD				SRI			
	Beta	95% CI Beta			Beta	95% CI		
		Lower	Upper	*R* ^2^		Lower	Upper	*R* ^2^
Current age	0.02	−0.08	0.012	0.001	−0.07	−0.18	0.05	0.006
Sex	0.11	−2.36	2.59	0.000	−1.12	−3.94	1.69	0.003
Race: Asian	−2.36	−8.08	3.36	0.007	−1.24	−7.81	5.33	0.008
Black/Africa American	−2.03	−6.57	2.52		−3.05	−8.28	2.18	
Other	−2.99	−8.71	2.72		−2.12	−8.69	4.45	
Rather not say	7.28	−1.07	15.63		0.47	−9.12	10.05	
Hispanic/Latino: Yes	−0.69	−5.22	3.84	0.015	−0.49	−5.69	4.71	0.000
Prefer not to answer	7.62	−0.72	15.95		0.00	−9.57	9.57	
Marital status	−1.92	−4.50	0.66	0.009	−2.23	−5.17	0.71	0.010
Employment status: Retired	−0.07	−3.68	3.54	**0.031**	−0.31	−4.44	3.81	**0.026**
**Unemployed**	**3.95**	**0.96**	**6.94**		**4.07**	**0.66**	**7.49**	
Income: < $49 K	3.55	−0.09	7.20	0.019	2.24	−1.93	6.40	0.015
$50 K–$150 K	1.35	−1.62	4.32		1.45	−1.94	4.85	
Prefer not to answer	−0.22	−3.96	3.53		−1.77	−6.05	2.51	
**Financial toxicity score**	**−0.30**	**−0.410**	**−0.19**	**0.120**	**−0.37**	**−0.49**	**−0.25**	**0.142**
Years from diagnosis	0.09	−0.08	0.25	0.001	−0.06	−0.25	0.13	0.002
Tumor grade: High	−1.46	−4.03	1.10	**0.022**	−1.06	−4.00	1.89	0.007
**None assigned**	**−7.90**	**−15.13**	**−0.66**		−4.94	−13.25	3.37	
Original extent: Biopsy	0.43	−3.81	4.67	0.003	−1.39	−6.22	3.44	0.003
Subtotal	−0.21	−2.99	2.56		−1.01	−4.17	2.16	
Resection NOS	1.63	−2.54	5.80		0.01	−4.74	4.76	
Number of sites involved	−0.54	−3.87	2.78	0.000	1.13	−2.66	4.91	0.002
On active treatment	−2.49	−5.70	0.71	0.010	−1.73	−5.40	1.94	0.004
Number of surgeries	−1.73	−4.20	0.75	0.008	−2.70	−5.51	0.12	0.015
Received radiation	−0.88	−4.02	2.25	0.001	−2.45	−6.01	1.11	0.008
Number of treatments	−0.56	−1.57	0.45	0.005	−0.09	−1.24	1.06	0.000
Recurrence: 1	0.05	−3.27	3.37	0.005	−4.15	3.41	−0.37	0.008
2 or more	−1.55	−4.60	1.50		−5.81	1.14	−2.34	
Disease progression on imaging	−0.62	−4.54	3.30	0.000	0.78	−3.69	5.25	0.001
KPS^a^	−0.10	−0.23	0.04	0.009	−0.13	−0.28	0.02	0.013
CCI age‐adjusted^b^	0.28	−1.07	1.62	0.001	−1.00	−2.49	0.049	0.010
Anticonvulsant use at visit^c^	0.72	−1.78	3.23	0.001	2.65	−0.18	5.47	0.015
**Psychotropic use at visit** ^c^	**5.16**	**2.47**	**7.85**	**0.061**	**4.67**	**1.58**	**7.75**	**0.039**

*Note:* Bolded values are significant at *p* < 0.05.

^a^ *N* = 215.

^b^ *N* = 180.

^c^ *N* = 221.

**FIGURE 2:**
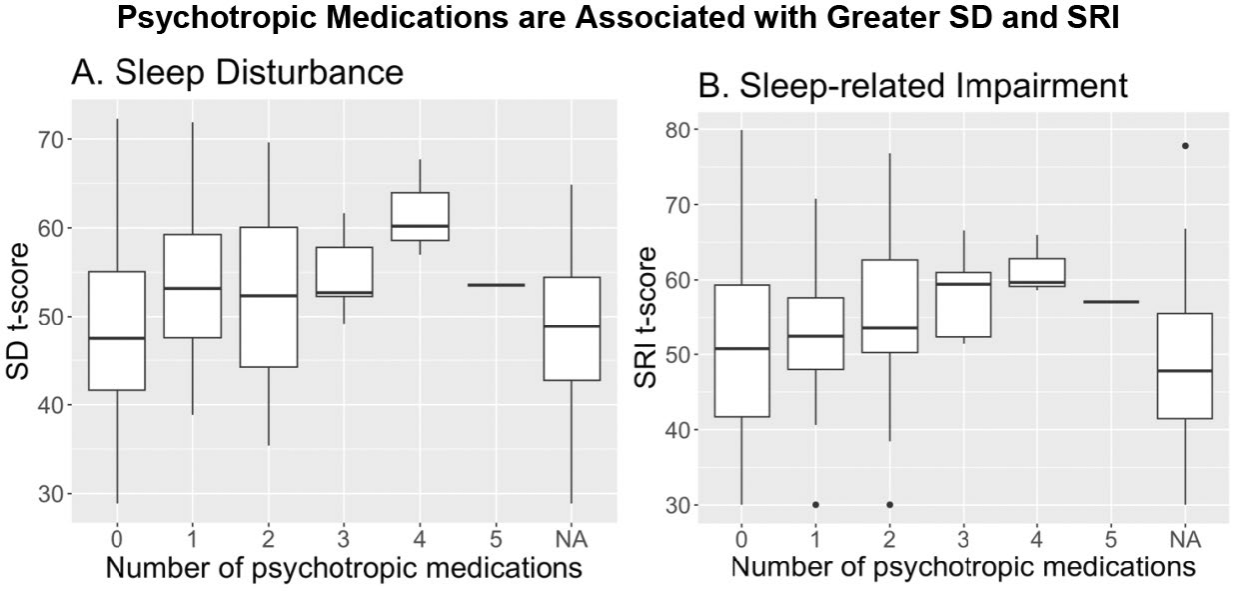
Impact of psychotropic medications on SD and SRI. Our analysis suggests that the more psychotropic medications a patient is taking, the more likely the patient will also report having greater SD and SRI.

Given that there are a variety of classes of psychotropic medications given for multiple clinical diagnoses and indications, we have included a breakdown of the psychotropic medications taken by participants in this sample (*n* = 152, Table S1). Indication for the prescribed medication was not collected from our participants, so that could not be accounted for in the analysis. Of note, 30 of the 61 participants taking psychotropic medications were taking more than 1, and 67% (16/24) of those taking benzodiazepines were taking additional psychotropic medications. It is possible that some individuals were taking benzodiazepines for sleep‐related issues; therefore, we repeated the analysis removing individuals who indicated they were taking benzodiazepines (*n* = 24). Both SD (*β* = 5.92, 95% CI [2.30, 9.54]) and SRI (*β* = 4.36, 95% CI [0.22, 8.51]) remain associated with psychotropic medication usage. Further, the more psychotropic medications a patient is taking, the greater the SD and SRI (Figure 2). The relationships with SD (*β* = 2.49, 95% CI [1.05, 3.93]) and SRI (*β* = 2.25, 95% CI [0.63, 3.86]) are also retained when participants taking benzodiazepines were removed from the analysis (SD (*β* = 3.37, 95% CI [1.24, 5.50]) and SRI (*β* = 2.92, 95% CI [0.50, 5.34])).

## 4 Discussion

Our results suggest that social determinants of health, specifically financial toxicity and employment status, may have a stronger impact on sleep–wake disturbances among PBT survivors than previous treatments or other clinical factors related to their disease course. This is significant because there is a distinct lack of published interventions focused on sleep for this population, and interventions focused on mitigating financial toxicity may help improve sleep quality [36, 37]. Treatment for cancer can induce financial toxicity or exacerbate existing financial difficulties. Direct medical costs for patients undergoing PBT treatment are high [38] and can be persistent for survivors as they remain on surveillance for disease recurrence or progression. The course of the disease or treatment can also prohibit patients from working, which was also associated with SD and SRI, further compounding financial difficulties. Addressing financial toxicity will require intervention on multiple levels, from the patient to insurers to policy. Smith and colleagues recommend a multidisciplinary approach to help patients navigate their cancer treatment journeys to minimize additional financial burden [37]. Studies investigating the impact of financial navigators on financial toxicity are ongoing in various cancer populations. Findings from these studies may be informative for developing patient‐level interventions in the PBT population.

Only 35 of our participants were diagnosed less than 1 year prior to NOB‐NHS entry. The SD and SRI surveys were completed at the 1‐year follow‐up, indicating all participants were diagnosed more than 1 year prior to completing the survey. Our previous work indicates that the incidence of SD is usually higher among patients at the time of diagnosis and for those on active treatment [3]. Given that most of our participants were not on active treatment (83%) and that the majority had undergone standard treatments such as radiation therapy, which is known to be associated with alterations in sleep, this may partially explain why clinical factors, such as radiation, chemotherapy, tumor grade, etc., were not found to be significant predictors of SD and SRI in our analysis. The impact of PBT treatment course on sleep may have waned over time, particularly because our survivors range from 1 to 37 years post‐diagnosis. Use of psychotropic medication was the only clinical factor associated with worse SD and SRI, although these medications had less impact than financial toxicity. Over a quarter of our participants were currently taking psychotropic medications, with the most common being selective serotonin reuptake inhibitors (*n* = 25) and benzodiazepines (*n* = 24). It is well known that these medications can impact sleep quality, with insomnia and hypersomnolence being the most common effects [39]. Patients may also be taking these medications for a variety of reasons; therefore, coordination of care to ensure mental health issues and medication side effects are well managed may help improve SD and SRI.

The incidence of SD (15%) and SRI (20%) among our participants approximated those observed in the general population and was less common than what is observed among PBTs at diagnosis or on active treatment [2]. This further supports factors that have been shown to be associated with SD and SRI in the general population, namely financial toxicity, unemployment, and psychotropic medication use, which were also most strongly associated with SD and SRI among PBT survivors. All clinical and tumor‐related factors had minimal effect on SD and SRI (*R* ^2^ ranged from 0.000 to 0.015), reinforcing the importance of assessing broader factors that impact patient symptoms among PBT patients and survivors. The observation that treatment‐related factors had a negligible effect on SD and SRI in our patients who are distal from intensive treatment has also been reported among a variety of cancer survivors [40, 41]. For example, in a study where Daniel and colleagues validated the pediatric PROMIS sleep measures in children being treated for cancer; they also reported that there was no difference in SD or SRI by diagnosis grouping or for those receiving radiation treatment [40]. Similar to Daniel's study, we also confirmed that the PROMIS‐SD and PROMIS‐SRI are reliable for evaluating sleep constructs among an adult PBT population.

Despite multiple studies indicating that the SD and SRI measures are reliable for assessing sleep–wake disturbances, others suggest the assumption that the SD measure is a unidimensional construct is incorrect. Brossoit and colleagues observed that several other research groups had posed similar hypotheses, but no consensus across studies was identified [42]. The mixed results were partially explained by different research approaches and various iterations of the PROMIS‐SD that were tested across studies. Their team also reports that the PROMIS measure is inconsistent with current theoretical understanding of sleep, which encompasses five dimensions that include duration, timing, satisfaction, efficiency, and alertness. Within the SD measure, they note that the items suggest measurement of two dimensions, satisfaction and efficiency, instead of a single dimension. When they tested their theory in six samples of working adults, their findings supported the 2‐factor hypothesis. It is possible that there are different predictors associated with each of these dimensions. For example, it is possible that financial toxicity impacted sleep efficiency, or symptoms of insomnia, whereas taking psychotropic medications may have had a greater impact on sleep satisfaction or perception of sleep quality. For future studies, it will be important to stay abreast of future iterations of the PROMIS measures in the event they are updated to better differentiate dimensions of sleep.

## 5 Study Limitations

This was a cross‐sectional sub‐analysis of an ongoing prospective study. On the NOB‐NHS, we enroll participants with over 100 different tumor types and participants who can join the study at any point during their disease trajectory. For many patients in this sample, SD and SRI data were collected well over a year past the primary point of diagnosis, and they were not on active treatment. Hence, our results are primarily pertinent to PBT survivors versus patients who are earlier in their disease course. Further, patients with different tumor types may have different levels of SD or SRI, which we are unable to disentangle with our diverse data set of patients with rare PBTs. Due to the diversity of tumor types and locations in the brain, as well as external risk factors assessed, we are unable to speculate on the biological mechanism that may lead to SD and SRI in this mixed patient population. In future studies, it will be important to consider identifying how financial toxicity and psychotropic medications may contribute to SD and SRI in this population.

These data remain important because neuro‐oncology providers treat diverse tumor types and there is scarce data about socioeconomic predictors of sleep problems in this population. Future prospective longitudinal studies should consider enrolling all participants at the point of diagnosis and assess SD, SRI, and financial toxicity over time to better assess which factors were present prior to the cancer diagnosis and which seem to worsen over the disease course. Given the prevalence of psychotropic medication use, it may be prudent for future sleep studies to collect these data, as well as the reason for medication use, to determine if SD and SRI are related to other comorbid conditions or to the effects of the medications themselves. Additionally, assessing the relationships of concurrent symptoms, such as distress, depression, and anxiety, may help to better differentiate if SD and SRI are more likely the result of other psychological disturbances, medication side effects, or both. In our analysis, we did not evaluate the impact of patient symptoms on SD and SRI, which will be a valuable future study.

## 6 Conclusion

Our study quantified the prevalence of SD and SRI among PBT survivors, established the validity of PROMIS sleep‐related instruments in this population, and identified several key predictors for sleep–wake disturbances. SD and SRI were most likely to be present in PBT survivors experiencing financial toxicity, unemployment, and the use of psychotropic medications. Our analysis of tumor‐related and clinical variables previously identified in the literature did not suggest a strong predictive relationship for SD and SRI among PBT patients. Future symptom research should include relevant social and clinical variables that have the potential to impact the outcome of interest. A more holistic assessment of factors that impact PROs may help identify meaningful intervention targets and improve quality of life for cancer survivors.

## Author Contributions

**Michelle L. Wright:** conceptualization (lead), investigation (equal), methodology (equal), project administration (lead), supervision (equal), visualization (lead), writing – original draft (lead), writing – review and editing (lead). **Jennifer Reyes:** data curation (equal), project administration (equal), writing – review and editing (equal). **Terri S. Armstrong:** conceptualization (equal), data curation (equal), funding acquisition (lead), investigation (equal), methodology (equal), project administration (equal), resources (lead), supervision (supporting), writing – review and editing (equal). **Bennett McIver:** data curation (equal), investigation (equal), writing – review and editing (equal). **Madhura Managoli:** data curation (equal), investigation (equal), writing – review and editing (equal). **Amanda L. King:** conceptualization (equal), data curation (equal), investigation (equal), methodology (equal), validation (equal), writing – original draft (equal), writing – review and editing (equal). **Zuena Karim:** data curation (equal), investigation (equal), writing – review and editing (equal). **Alvina A. Acquaye‐Mallory:** data curation (equal), project administration (equal), writing – review and editing (equal). **Brayden Chavis:** data curation (equal), writing – review and editing (equal). **Hope Miller:** data curation (equal), formal analysis (equal), investigation (equal), validation (equal), writing – review and editing (equal). **Elizabeth Vera:** conceptualization (equal), data curation (lead), formal analysis (lead), investigation (equal), methodology (lead), supervision (equal), validation (equal), writing – review and editing (equal). **Tricia Kunst:** data curation (equal), investigation (equal), project administration (equal), validation (equal), writing – review and editing (equal). **Morgan Johnson:** data curation (equal), investigation (equal), writing – review and editing (equal). **Anna Choi:** data curation (equal), investigation (equal), project administration (equal), writing – review and editing (equal). **Ewa Grajkowska:** data curation (equal), formal analysis (equal), validation (equal), writing – review and editing (equal).

## Ethics Statement

Neuro‐Oncology Branch Natural History Study (NOB‐NHS; NCT #: NCT02851706; PI: T.S. Armstrong) was approved by the NIH Institutional Review Board.

## Conflicts of Interest

The authors declare no conflicts of interest.

## Supporting information


Table S1.

## Data Availability

The data that support the findings of this study are available on request from the corresponding author. The data are not publicly available due to restrictions (e.g. their containing information that could compromise the privacy of research participants).
